# Access to context-specific lexical-semantic information during discourse tasks differentiates speakers with latent aphasia, mild cognitive impairment, and cognitively healthy adults

**DOI:** 10.3389/fnhum.2024.1500735

**Published:** 2025-01-22

**Authors:** Brielle C. Stark, Sarah Grace Dalton, Alyssa M. Lanzi

**Affiliations:** ^1^Department of Speech, Language and Hearing Sciences, Indiana University Bloomington, Bloomington, IN, United States; ^2^Program in Neuroscience, Indiana University Bloomington, Bloomington, IN, United States; ^3^Department of Communication Sciences and Special Education, University of Georgia, Athens, GA, United States; ^4^Department of Communication Sciences and Disorders, University of Delaware, Newark, DE, United States; ^5^Delaware Center for Cognitive Aging Research, University of Delaware, Newark, DE, United States

**Keywords:** aphasia, language, narrative, discourse, mild cognitive impairment

## Abstract

**Purpose:**

Mild language impairments experienced by adults with neurogenic communication disorders are often difficult to detect due to the lack of sensitive traditional performance-based measures. This is problematic since many adults who have mild language deficits experience daily activity and participation limitations that are undetected and not managed. This study evaluates the potential for variables derived through core lexicon analysis to differentiate two clinical groups (latent aphasia, MCI) from each other, and from a cognitively healthy adult group, across three different discourse tasks (Aim 1). Innovatively, it also contrasts the sensitivity with which each task differentiates the groups based on this metric (Aim 2).

**Methods:**

Transcribed connected speech data from TalkBank were analyzed for three discourse tasks (i.e., Sandwich Procedure, Cat Rescue Picture Description, and Cinderella Story) from three participant groups [Mild Cognitive Impairment (MCI) *n* = 30, stroke-induced latent aphasia *n* = 29, and Cognitive Healthy Adults (CHA) *n* = 56]. Aim 1 used one-way ANOVAs (or non-parametric equivalents) to identify differences in lexical variables (total number of core lexical items; proportion of core lexical items out of all words produced; and rate of core lexical items produced per second) between participant groups. Aim 2 used linear discriminant analysis with cross validation to characterize the sensitivity of discourse task in identifying lexical variables differentiating the participant groups.

**Results:**

Univariate analysis revealed significant differences among the three participant groups. During the Cinderella task, the latent aphasia and MCI groups produced significantly fewer core lexical items than CHAs, while their proportion of core lexical items to total tokens was higher than CHAs. The latent aphasia group produced core lexical items more slowly than the MCI group for all three discourse tasks. Finally, individuals with latent aphasia produced significantly fewer core lexical items during the Sandwich task than either the MCI or CHA groups. Aim 2’s sensitivity analysis revealed that number of core lexical items produced during the Cinderella task best differentiated the MCI group from CHAs, number of core lexical items produced during Sandwich best differentiated latent aphasia from CHAs, and core lexical items per second during Cinderella best differentiated latent aphasia from MCI.

**Conclusion:**

Our study suggests that the Cinderella story is more sensitive than a picture description task for demonstrating the subtle lexical-semantic changes in MCI and latent aphasia compared to CHAs. Core lexicon appears to be a sensitive discourse metric to identify linguistic differences between CHAs and individuals with mild cognitive and/or language deficits. These findings further support calls to provide speech/language and cognitive therapy to individuals with MCI and/or latent aphasia.

## Introduction

People with very mild language impairments frequently experience significant activity and participation limitations (Cavanaugh and Haley, 2020). These very mild language impairments are often not discernable using traditional performance-based neuropsychological tests (e.g., standardized language and cognitive norm-referenced measures), which lack adequate sensitivity. However, it is important that these individuals receive speech/language services to reduce the negative impacts of their language impairment Referrals cannot happen, and adequate treatment plans cannot be made, unless there are metrics sensitive enough to detect mild impairments. Two clinical groups that are at particular risk of having unmet rehabilitation needs include individuals with mild cognitive impairment (MCI) and individuals with latent aphasia following stroke.

MCI is a clinical syndrome characterized by cognitive decline that is greater than expected for an individual’s age and education level but does not significantly interfere with daily functioning (American Psychiatric Association, 2013). Language impairments in MCI can manifest in various ways such as reduced verbal fluency (Weakley et al., 2013) or errors in confrontation naming (Ahmed et al., 2013). In older adults, language decline may be indicative of underlying neurodegenerative diseases, such as Alzheimer’s disease, and may predict progression to further cognitive impairment earlier than memory decline (Mueller et al., 2018b; Oulhaj et al., 2009). Diagnosing someone early in the disease process is not only difficult due to the lack of sensitive measures (Petersen and Yaffe, 2020) but also the expertise and time needed for conducting a comprehensive evaluation (Alzheimer’s Association, 2022). For example, a recent study found that the *Montreal Cognitive Assessment* (MoCA; Nasreddine et al., 2005), a common cognitive screening tool used by speech-language pathologists (SLPs) (Lanzi et al., 2023; Roitsch et al., 2021) does not have adequate sensitivity or reliability to detect mild language/communication challenges in adults with MCI (Stagge et al., 2024). Early detection and monitoring of language impairments in MCI is especially crucial for appropriate intervention and management strategies, e.g., early uptake of lifestyle management strategies (Chandler et al., 2019; Clark et al., 2005; Cohen et al., 2021; Ropacki et al., 2017; Sabbagh et al., 2020).

The second group at increased risk of unmet rehabilitation needs are individuals who have experienced a left hemisphere stroke but score above the cut-off for aphasia on standardized language assessments. These individuals have historically received little attention in the research literature, either by being excluded for not having aphasia or by being grouped with individuals with anomic aphasia (e.g., Cruice et al., 2014; Papanicolaou et al., 1988; Sekine and Rose, 2013; Sekine et al., 2013). However, increased attention has been paid to this group recently (Crutch and Warrington, 2003; Cunningham and Haley, 2020; Dalton and Richardson, 2015, 2019; DeDe and Salis, 2020; Fromm et al., 2017; Law et al., 2015; Martzoukou et al., 2023; Richardson et al., 2018, 2021; Salis and DeDe, 2022; Silkes et al., 2020). The terminology used to refer to this group has varied widely, but there appears to be a coalescence in the literature around the term “latent aphasia” (e.g., DeDe and Hoover, 2021; DeDe and Salis, 2020; Martzoukou et al., 2023; Salis and DeDe, 2022; Silkes et al., 2020; Zhang et al., 2024). We prefer this label since (1) it does not refer to a specific clinical assessment (in contrast to another common label, “not aphasic by WAB [Western Aphasia Battery]”); and (2) the language impairments observed in this group are consistent with the definition of latent as a “quality or state existing but not yet manifest” and “lying dormant or hidden until circumstances are suitable for development or manifestation” (Oxford Languages). Individuals with latent aphasia produce discourse that is significantly different from healthy controls who have not experienced a stroke. For example, individuals with latent aphasia demonstrate reduced typicality and informativeness, fewer utterances, lower lexical diversity and lexical entropy, reduced efficiency, longer formulation time, longer silent pauses, and slower speech rate than healthy controls (e.g., Cunningham and Haley, 2020; Dalton et al., 2020a; DeDe and Salis, 2020; Fromm et al., 2017). Continued investigations that focus on improving identification of latent aphasia are warranted given these findings.

### Discourse and lexical access

Discourse, or language production beyond the sentence level produced for a specific purpose (Armstrong, 2000), is a fundamental component of communication. Successful discourse production relies upon a complex interplay between cognitive and linguistic processes. It comprises microlinguistic (e.g., syntax, phonology, lexical-semantics) and macrostructural components (e.g., topic management, story grammar) (Dipper et al., 2021), as well as cognitive functions such as semantic memory (Tchakoute et al., 2017) and executive function (Cannizzaro and Coelho, 2013; Dutta et al., 2024). Because of the long history of eliciting these more complex communication behaviors in patients with dementia and stroke, many different terms have been used, including “connected speech,” “discourse,” “language,” or “connected language.” Connected speech is perhaps the most frequently used term and highlights the historical focus on microlinguistic measures of production (such as type/token ratio, words per minute and prosodic factors), to the exclusion of more complex language (e.g., inferencing, organization, and sequencing) or cognitive (e.g., judgment, reasoning, problem-solving) processes. We use the term “discourse” since successful communication at this level requires speech, language, and cognitive processes working in tandem.

Discourse provides complementary and extended information to that collected through typical neuropsychological performance-based language measures (e.g., verbal fluency tasks, confrontation object naming). Research suggests that discourse analysis may be sensitive to early neuropsychological changes during the MCI phase, in subjective cognitive decline, and in subtle presentations of language impairments after brain injury (Ahmed et al., 2013; Berisha et al., 2015; Garrard et al., 2005). Of particular interest to the current study is that discourse analysis may sensitively demonstrate language changes in MCI from those associated with cognitively healthy aging, as well as to differentiate whether language is ‘sufficiently’ impaired post-stroke to warrant a referral for speech-language therapy services.

While discourse can be both interactional and monologic, the predominant focus in clinical practice and research has been on monologic discourse. Within monologs, single picture description tasks are the predominant means of elicitation for both individuals with MCI (Filiou et al., 2020; Mueller et al., 2018a) and those with aphasia (Bryant et al., 2016). Single picture description tasks are included in most standardized assessment batteries for individuals with communication impairments (e.g., Goodglass and Kaplan, 1972; Kertesz, 2007; Wilson et al., 2018). In this type of task, a relatively complex visual scene is presented to the client, and they are asked to talk about it. Importantly, specific elicitation instructions vary (e.g., with some tasks specifying to “speak in full sentences,” and others lacking precise instructions), which previous research shows may have an impact on discourse production (Wright and Capilouto, 2009). In the MCI population, it has been argued that using single picture descriptions minimizes demands on potentially impaired memory systems and more sensitively demonstrates language, rather than memory, difficulties (Mueller et al., 2018a). Giving someone a picture to describe constrains the language that can be produced, therefore requiring individuals to access specific vocabulary to successfully describe the picture. This restriction on the ‘correct’ vocabulary can demonstrate phonological and lexical-semantic impairments, in that persons with stroke or MCI may be unable to produce the relevant vocabulary, produce unrelated or vague vocabulary (e.g., “that one”), or produce various errors (e.g., “dog” instead of “lion”). Despite these strengths, single picture description tasks also have drawbacks. They tend to require little cognitive effort and therefore may elicit language that does not demonstrate subtle clinical changes. For example, in a group of persons with and without aphasia, a single picture description was shown to demonstrate lower propositional idea density (e.g., fewer semantically relevant words) than a narrative task (retelling a well-known story) (Stark, 2019) which poses a problem for documenting changes in MCI. Indeed, propositional idea density has been widely examined in studies of dementia and aging (Kemper et al., 2001; Snowdon, 1996), with individuals at risk for cognitive decline or Alzheimer’s disease showing a decline in propositional idea density and syntactic complexity. Finally, a recent study investigated the utility of discourse variables derived from a single picture description task to differentiate between cognitively healthy adults and individuals with MCI (Mefford et al., 2023). Results indicated that this task had variable sensitivity to group differences by MCI subtype (amnestic vs. non-amnestic) and/or by the proportion of various subtypes in an undifferentiated MCI population (Mefford et al., 2023).

Other common ways of collecting discourse samples include the retelling of fictional, well-known (“familiar”) narratives. The person is typically presented with a wordless picture book (e.g., Cinderella), and asked to retell the story with any information they knew about the story beforehand and what they had just seen in the book (MacWhinney et al., 2010; Saffran et al., 1989). Another common method involves the description of a procedure, where the participant is asked to tell the listener “how to do” something. Procedural narratives are particularly interesting for demonstrating communicative competence via multimodal communication, because these narratives typically produce spatial language that describes the position, relationship, and movement of objects (likely because they draw on implicit, motor memory) and also associate with co-speech, meaningful gesture (Pritchard et al., 2015; Stark and Cofoid, 2022; Stark and Oeding, 2024).

### Discourse analysis

Early detection and monitoring of language impairments in MCI is especially crucial for appropriate intervention and management strategies (Chandler et al., 2019; Cohen et al., 2021; Ropacki et al., 2017; Sabbagh et al., 2020). Further, persons with stroke (who may also have latent aphasia) are at a heightened risk for development of MCI (e.g., Sachdev et al., 2009) and dementia (Kuźma et al., 2018). It is therefore extremely important to sensitively monitor for transition from a primarily stable clinical state (e.g., chronic stroke-induced latent aphasia) to a progressive clinical state (e.g., chronic stroke-induced aphasia and dementia diagnosis).

Burgeoning research has demonstrated that individuals with latent aphasia produce discourse distinct from healthy control speakers and speakers with anomic aphasia during narrative discourse. Individuals with latent aphasia make more word errors, speak more slowly, and produce decreased essential content compared to cognitively healthy peers (Fromm et al., 2017). Others have also found increased silent pause duration in individuals with latent aphasia during narrative tasks (DeDe and Salis, 2020). Growing research suggests that there are measurable differences in specific microlinguistic processes (e.g., fluency and semantics) between cognitive healthy adults and clinical syndromes from Alzheimer’s disease (Filiou et al., 2020; Mueller et al., 2018b), though differentiation in discourse-level (e.g., coherence) features of MCI from cognitively healthy aging adults has seen mixed results (Bschor et al., 2001; Toledo et al., 2018). These mixed finding may be the result of the majority of analyses only evaluating a single picture description task that elicits a relatively short language sample (e.g., Cookie Theft) (e.g., Lanzi et al., 2023; Mueller et al., 2018b). Therefore, there is a distinct gap in knowledge about the extent to which different discourse tasks can be leveraged to differentiate persons with clinical diagnoses that present with subtle language impairments (latent aphasia; MCI) from cognitively healthy adults.

While clinicians and researchers recognize the importance of discourse analysis and report a desire to use it (Bryant et al., 2017; Cruice et al., 2020; Stark et al., 2021), it is not standard clinical (or research) practice because a variety of barriers exist, e.g., lack of training, lack of tools, and lack of time (Stark et al., 2021; Stark and Dalton, 2024). Transcript-based analysis is the gold standard for comprehensively analyzing discourse, but transcribing at a detailed level is rarely feasible in clinical settings, and the level of detail contained in research-setting transcripts (e.g., phonetic analysis) may not be necessary for clinical decision making. For this reason, metrics that are easy to extract (either from a quick transcript or perceptually) are ideal in clinical settings (Stark and Dalton, 2024).

### Core lexicon analysis

Core lexicon analysis is a discourse metric that evaluates context-specific access to lexical-semantic knowledge (Dalton et al., 2020a,b). For a given discourse task, a core lexicon is comprised of typical lexical items produced by cognitively healthy persons (Dalton et al., 2020a,b; Kim and Wright, 2020). A unique aspect of core lexicon analysis compared to similar measures, such as correct information units (Nicholas and Brookshire, 1993), is the focus on the typicality of vocabulary used. For example, during the Cinderella task, cognitively healthy controls tend to use lexical items such as “prince” and “slipper,” to describe core components of the story (Dalton et al., 2020a,b). Using less specific vocabulary such as “man” or “shoe” in place of these core lexical items would still be broadly informative but leads to a less rich and nuanced discourse production. Indeed, core lexicon measures have been shown to correlate with main concept production, suggesting that single lexical items can reflect broader ‘main ideas’ (sometimes called ‘macropropositions’) constructed during discourse (Dalton and Richardson, 2015). As such, core lexicon analysis provides an interface between linguistic and propositional levels of discourse.

Recent work has suggested that evaluating core lexical items produced during discourse may be a sensitive way to evaluate longitudinal change from acute to chronic stage in post-stroke aphasia (Kim et al., 2022) and has suggested that core lexicon production associates with dementia severity in persons with Alzheimer’s disease (Kintz et al., 2024). Indeed, the subtle change in the ability to access topic-relevant lexical-semantic vocabulary has been documented in persons with Alzheimer’s disease (Mueller et al., 2020) and latent aphasia (Dalton and Richardson, 2015), though not yet systematically characterized across discourse tasks or across clinical groups. More recent work from Chen et al. (2024) suggests that core lexical access, in particular, is the most sensitive in differentiating language between individuals with MCI and cognitively healthy adults. This study evaluated *n* = 16 English-speaking participants with MCI and *n* = 16 matched cognitively healthy adults, examining eight lexical-semantic features across four discourse tasks (two picture descriptions, a familiar story narrative [Cinderella], and a procedural narrative) (Chen et al., 2024). Univariate analyses showed inter-group differences in core lexicon and other variables, depending on the task. Multivariate pattern analysis demonstrated that the Cinderella narrative was the only task that discriminated the two groups above chance (65.6%), and when examining the lexical-semantic features that drove this, identified that the MCI group produced fewer core lexical items. The authors suggest that this finding shows that individuals with MCI exhibit a decrease in lexical diversity and richness in a story recall setting.

While both individuals with MCI and individuals with latent aphasia present with lexical-semantic impairments behaviorally, it is important to consider the underlying cognitive changes driving performance between the groups. For individuals with latent aphasia, the lexical-semantic impairment is likely a result of difficulty accessing mental representations of the lexicon, although mental representations remain intact. On the other hand, lexical-semantic impairment in individuals with MCI may arise via several routes. For individuals with amnestic MCI, lexical-semantic impairments may be a result of deficits in working or semantic memory. For individuals with non-amnestic MCI, lexical-semantic impairments may be driven by executive function, memory, or language deficits.

Core lexicon lists have been developed for discourse tasks commonly used to evaluate clinical samples (Dalton et al., 2020a,b). Of particular note and the main impetus of the current study is that burgeoning evidence supports the feasibility and utility of core lexicon analysis in clinical settings (Dalton et al., 2020a,b; Kim and Wright, 2020). Preliminary research suggests that, once clinicians are familiar with the core lexical item checklists for the discourse samples that they typically utilize, scoring can be completed without transcripts and potentially in real time (Dalton et al., 2020a,b). Given this high potential for clinical utilization, additional investigations of core lexicon’s sensitivity to group differences are warranted.

### Motivation and study aims

This study evaluates the potential with which variables derived through core lexicon analysis can differentiate two clinical groups (latent aphasia, MCI) from each other, and from a cognitively healthy adult group, across three different discourse tasks (Aim 1). Innovatively, it also contrasts the sensitivity with which each task differentiates the groups based on this metric (Aim 2).

## Methods

Methods details were guided by the best practices for publishing on spoken discourse in aphasia (Stark et al., 2022); a table with page numbers highlighting each best practice can be found in the Supplementary material.

### Participants

All participants were drawn from the English corpora of the TalkBank consortium, specifically, DementiaBank (Lanzi et al., 2023) and AphasiaBank (MacWhinney et al., 2011). Authors BCS and AL directly contributed data: BCS to the latent aphasia group and cognitively healthy adult group (The NEURAL Research Lab Corpuses in AphasiaBank, ethical approval from Indiana University), and AL to the MCI group and cognitively healthy adult group (The Delaware Corpus in DementiaBank, ethical approval from the University of Delaware). All other data came from the consortium, which were provided by authors who had their own established IRBs.

Participant groups were relatively matched for age, gender, education, and race/ethnicity, as seen in Table 1 which reports participant demographic and neuropsychological information. Thirty individuals with MCI, 27 with Latent Aphasia, and 56 Cognitive Healthy Adults were ultimately included.

**Table 1:** Demographic and neuropsychological information for included participants.

	MCI (*N* = 30)	Latent aphasia (*N* = 27)	CHA (*N* = 56)	Statistics
Age (yrs)				
Mean (SD)	70.3 (5.50)	66.6 (9.00)	69.0 (6.38)	*F*(2,110) = 2.18, *p* = 0.12
Median [Min, Max]	70.0 [61.0, 79.0]	68.0 [51.2, 78.4]	68.9 [51.0, 79.8]	
Sex				
Female	16 (53.3%)	15 (55.6%)	32 (57.1%)	*X*^2 = 0.12, df = 2,^ *p* = 0.94
Male	14 (46.7%)	12 (44.4%)	24 (42.9%)	
Education (yrs)				
Mean (SD)	16.1 (1.81)	16.4 (3.12)	16.2 (2.34)	*F*(2,110) = 0.11, *p* = 0.898
Median [Min, Max]	16.0 [14.0, 20.0]	16.0 [12.0, 22.0]	16.0 [12.0, 21.0]	
Race/Ethnicity				
African American/Black	3 (10%)	1 (3.7%)	3 (5.4%)	*X*^2 = 3.14, df = 4,^ *p* = 0.53
White, non-Hispanic	27 (90%)	26 (96.3%)	51 (91.1%)	
Hispanic or Latinx	0 (0%)	0 (0%)	2 (4.6%)	
Western aphasia battery (aphasia quotient)				
Mean (SD)	Not collected	96.3 (1.85)	Not collected	None computed
Median [Min, Max]	Not collected	95.8 [93.8, 100]	Not collected	
Montreal cognitive assessment*				
Mean (SD)	24.0 (2.67)	Not collected	26.8 (1.48)	*F*(1,69) = 32.57, *p* < 0.0001
Median [Min, Max]	24.0 [19.0, 28.0]	Not collected	26.0 [24.0, 29.0]	
Mini mental state exam (unadjusted)*				
Mean (SD)	Not collected	Not collected	28.0 (1.56)	None computed
Median [Min, Max]	Not collected	Not collected	[25.0, 30.0]	

MCI, Mild Cognitive Impairment; CHA, Cognitively Healthy Adults.

*Montreal cognitive assessment and mini mental state exam are two cognitive screeners collected for the MCI group and for the cognitively healthy peer group. Original labs collecting the data collected one or the other for the cognitively healthy group, so not all persons within that group have data for both assessments.

#### Inclusion

For homogeneity of age, and to reflect typical diagnostic ranges of stroke aphasia and MCI, individuals were included if they were aged 50–79 years.

*Individuals with latent aphasia*—defined as testing above a standard aphasia cut off on a standardized battery [the *Western Aphasia Battery-Revised* or *Western Aphasia Battery Bedside* (Kertesz, 2007)]—were identified across all corpora in the AphasiaBank database. All had impacted language as a result of a stroke; some individuals had more than one stroke, but the number of strokes (and location of stroke) was not always reported. Motor speech information was limited for participants.

*Individuals with amnestic MCI* were identified through the Delaware Corpus of DementiaBank. Amnestic MCI status was documented in the database and was based on the National Institute on Aging and Alzheimer’s Association working group (Albert et al., 2011) and Petersen (2004) criteria. That is, individuals with amnestic MCI produced at least one cognitive test score 1.5 standard deviations below age-and-demographically matched cognitively healthy adults but were functionally independent as measured by the *Clinical Dementia Rating Scale* obtained from a structured interview with a study partner (Morris, 1993). Since all participants presented with amnestic MCI, the groups’ primary cognitive deficits were with memory function.

*Cognitively healthy adults* (CHA) were selected from the control samples within DementiaBank and AphasiaBank, across various corpora. Potential participants were selected based on the age and sex distribution of the two clinical groups. All cognitively healthy adults had either a Mini Mental State Exam or Montreal Cognitive Assessment score in the typical range.

#### Exclusion

Individuals were excluded from the analysis if they did not have language data for the three tasks of interest, described below, or were otherwise missing demographic or neuropsychological testing data.

### Elicitation materials and database

Transcribed speech data were already available in AphasiaBank and DementiaBank for participants described above. These transcriptions undergo reliability verification prior to inclusion in the database, though the methodology differs by the contributing lab. The general process is that each contributing lab completes in-house reliability checks, then sends transcription and audio or video data to the TalkBank team, who then double-check the transcriptions for accuracy.

Transcribed speech data were analyzed for three discourse tasks: Cat Rescue description (a single picture description), the Cinderella narrative (a familiar, fictional story retell with no pictorial cues during the retelling), and the Sandwich narrative (a procedural narrative where individuals tell how to make a peanut butter and jelly sandwich with no pictorial cues). Transcriptions were available in AphasiaBank and DementiaBank and were not further checked for the purposes of this study since previous research has reported high fidelity of transcription (Dalton and Richardson, 2015). The transcriptions contained orthographic and phonetic information and were coded using Codes for the Human Analysis of Transcripts (CHAT) (MacWhinney, 2000). For full task instructions and pictures given to participants, see the AphasiaBank and DementiaBank protocols, located on their respective websites. Media and transcripts for all participants are available through consortium membership to AphasiaBank and DementiaBank.

### Dependent variable summary

Core lexical analysis is a clinically feasible tool for evaluating lexical-semantic, context-specific knowledge and has been used to evaluate a variety of different spoken discourse prompts and procedures in aphasia (Dalton et al., 2020a,b; Dalton and Richardson, 2015; Kim et al., 2022; Kim and Wright, 2020) and dementia (Kintz et al., 2024). In this study we used previously established core lexicon checklists for the Cat Rescue, Cinderella, and Sandwich discourse tasks (Dalton et al., 2020a,b).

As such, core lexical information was modeled in three ways: (1) raw number of core lexicon items produced, reflecting topic-relevant lexical-semantic access; (2) core lexicon items as a proportion of total tokens (similar to a metric of lexical diversity but with an emphasis on typicality), reflecting the extent to which the information produced was topically informative; and (3) rate of core lexicon items produced, modeled per second of speech, reflecting the extent to which topic-relevant lexical-semantic information was produced in an efficient manner.

These three variable iterations were chosen because of their potential clinical feasibility. Studies have demonstrated that the number of core lexical items is able to be collected in real time for the elicitation materials used in our study (Dalton et al., 2020a,b; Kim and Wright, 2020) and the proportion of core lexical items and core lexical items per second can be easily calculated *post hoc* by dividing the total core lexical items by total words and total seconds spoken. It is common in clinical and research settings to record spoken discourse in order to do analyses later (Bryant et al., 2017; Stark et al., 2021), so ascertaining these variables is an additional step, but straightforward. Further, the efficiency of discourse production (modeled as variables per second or minute) has been before shown to be a sensitive measure across other variables, like correct information units (Boyle et al., 2022; Doyle et al., 1995; Nicholas and Brookshire, 1993; Stark et al., 2023).

### Extracting lexical variables from transcripts using CLAN

While core lexical items can be scored by hand in a clinic setting (Dalton et al., 2020a,b), this analysis opted to automatically extract them from the pre-created transcripts (Dalton et al., 2022). The Computerized Language Analysis Program (CLAN) program was used to automatically extract the dependent variables described above (MacWhinney, 2000). CLAN version 19jul23 on Windows was used. Table 2 describes the list of CLAN commands that were run on the transcripts for all participants.

**Table 2:** CLAN commands to extract lexical dependent variables.

CLAN command	Description
mor *.cha	Assign morphological and grammatical tier to the transcribed language, for all CHAT files in folder (*.cha)
Eval + t*PAR + u + g”NAME” + n *.cha	Run the EVAL command, which generates a variety of linguistic information (e.g., duration of speech sample and total tokens) for each discourse task at a time (+g”NAME”). Run this command only for the speaker (+t*PAR), not the experimenter. Merge all files into a spreadsheet (+u). Run for all CHAT files in folder (*.cha). Note that this analysis automatically ignores all excluded, off-topic utterances, as well as repeated or revised words and phrases.
corelex + lNAME + t*PAR-s“ < + exc > ”+f + u *.cha	Run the CORELEX command, which generates the core lexical items for a specific discourse task (+lNAME, e.g., “lCinderella”). Run this command only for the speaker (+t*PAR), not the experimenter. Ignore any excluded utterances (−s” < + exc>”). Merge all files into a spreadsheet (+u) and send to file (+f). Run for all CHAT files in folder (*.cha).

### Analysis

All analyses were run using RStudio 2023.12.1 Build 402 and R version 4.3.2 (2023-10-31 ucrt). Project analyses and de-identified data are available on the Open Science Framework.^1^

#### Aim 1: identify differences in lexical variables between participant groups

It was determined that measures did not conform to linear ANOVA assumptions (via Bartlett and Levene’s tests), and thus a Brown-Forsythe one-way ANOVA for unequal variances was used (via R package *onewaytests*). To run this analysis, an interaction term between Group and Task was created. Then, the interaction term was modeled alongside each dependent variable. If the ANOVA was significant, a *post hoc* Games Howell Test was employed via *rstatix* package to examine significant pairwise comparisons. The post hoc Games Howell Test took into account multiple comparisons.

To evaluate the actual core lexical items produced by participants of each group, one-way ANOVAs (or non-parametric equivalents) were run between the core lexical items (the number varied by task) across groups. This enabled us to evaluate the impact of the participant group on the percentage of group members producing an item from each task’s core lexicon at least once. To identify the achieved power of each one-way analysis, a sensitivity analysis was computed for each variable using harmonic means to account for different sample sizes per group per task and the Cohen’s f from each ANOVA.

#### Aim 2: task sensitivity in identifying lexical variables differentiating the three participant groups

The second primary analysis evaluated the extent to which dependent variables enabled supervised classification into participant groups, by task. Latent discriminant analysis using the *MASS* and *caret* packages in R was employed. To avoid overfitting, 50% cross validation was used (i.e., the model was trained on 50% of data and then tested on 50% of the remaining, unseen data). Of interest was the ability of the dependent variables to differentiate between the clinical groups (latent aphasia, MCI), and between the clinical groups and non-clinical group (latent aphasia, CHA; MCI, CHA). Latent discriminant analysis was only conducted for pairwise comparisons that were indicated to be significantly different in Aim 1. As is best practice, prediction accuracy is provided for each classification. The current study was not pre-registered.

## Results

### Aim 1: identify differences in lexical-semantic measures between subject groups

**Figure 1:**
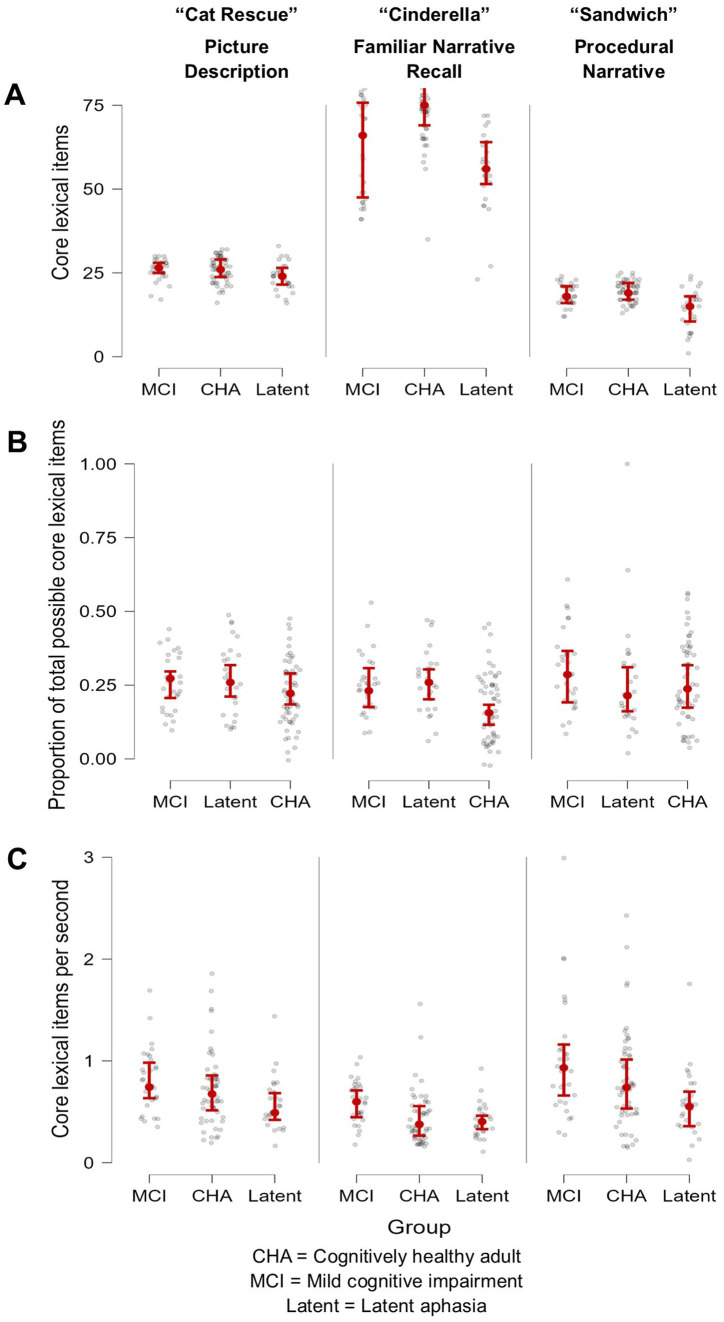
Visual demonstration of three core lexical variables, arranged by participant group and task. **(A)** Number of core lexicon items produced was most successful at differentiating the MCI group from the CHA group but only for the overlearned narrative Cinderella task, where the peer group produced a higher quantity of lexical-semantic information; **(B)** Proportion of core lexicon items was most successful in differentiating the MCI and the latent aphasia groups from the CHA group, but only for the overlearned narrative Cinderella task; and **(C)** Core lexical items produced per second was most successful at differentiating the latent aphasia from the MCI group across all tasks, where the latent aphasia group produced lexical-semantic information less fluently.

Brown-Forsythe one-way test (alpha = 0.05) indicated a significant difference across tasks and groups for the number of core lexicon items (*F*[8, 114.74] = 313.92, *p* < 0.00001, Cohen’s *f* = 2.89, achieved *β* > 0.99), proportion of core lexicon to total tokens (*F*[8, 162.61] = 5.02, *p* = 0.0001, Cohen’s *f* = 0.36, achieved *β* = 0.999), and core lexicon items per second (*F*[8, 179.94] = 11.71, *p* < 0.000001, Cohen’s *f* = 0.52, achieved *β* > 0.99). Therefore, Games-Howell post hoc tests with multiple comparison correction were performed for all dependent variable comparisons to evaluate significant pairwise differences between participant groups and for each task, described below. See Table 3 for full descriptive statistics of variables used in the one-way tests, and Table 4 for post hoc analyses. See Figure 1 for a visual comparison of significant variables across groups.

**Table 3:** Summary of performance across dependent variables, by group and task.

Latent aphasia (*N* = 27)					MCI (*N* = 30)			CHA (*N* = 56)	
Task	*Cat rescue*	*Cinderella*	*Sandwich*	*Cat rescue*	*Cinderella*	*Sandwich*	*Cat rescue*	*Cinderella*	*Sandwich*
Core lexicon items (number)									
Mean (SD)	24.0 (4.19)	56.4 (13.4)	14.3 (6.04)	25.8 (3.24)	62.6 (14.5)	18.6 (3.34)	25.9 (3.70)	75.6 (10.7)	19.5 (2.90)
Median [Min, Max]	24.0 [16.0, 33.0]	56.0 [23.0, 88.0]	15.0 [1.00, 24.0]	26.5 [17.0, 30.0]	66.0 [41.0, 81.0]	18.0 [12.0, 24.0]	26.0 [16.0, 32.0]	75.0 [35.0, 91.0]	19.0 [13.0, 25.0]
Core lexicon items as a proportion of tokens									
Mean (SD)	0.273 (0.098)	0.265 (0.092)	0.269 (0.190)	0.260 (0.0703)	0.243 (0.0837)	0.295 (0.127)	0.238 (0.0901)	0.164 (0.0728)	0.251 (0.130)
Median [Min, Max]	0.259 [0.093, 0.475]	0.259 [0.050, 0.469]	0.214 [0.010, 1.00]	0.273 [0.137, 0.447]	0.231 [0.0914, 0.402]	0.286 [0.0773, 0.600]	0.222 [0.0785, 0.473]	0.156 [0.0529, 0.371]	0.238 [0.0535, 0.567]
Core lexicon items per second									
Mean (SD)	0.575 (0.252)	0.423 (0.156)	0.589 (0.327)	0.818 (0.299)	0.595 (0.207)	1.03 (0.586)	0.728 (0.352)	0.434 (0.253)	0.808 (0.466)
Median [Min, Max]	0.491 [0.200, 1.45]	0.404 [0.123, 0.917]	0.552 [0.017, 1.75]	0.743 [0.384, 1.70]	0.598 [0.224, 1.04]	0.933 [0.270, 3.00]	0.675 [0.219, 1.86]	0.378 [0.165, 1.57]	0.740 [0.152, 2.43]
Missing	0 (0%)	0 (0%)	0 (0%)	0 (0%)	0 (0%)	0 (0%)	0 (0%)	1 (1.8%)	0 (0%)

MCI, Mild Cognitive Impairment; CHA, Cognitively Healthy Adults.

**Table 4:** Games-Howell post hoc test, using adjusted *p*-values that account for multiple comparisons.

Task	Cat rescue			Cinderella			Sandwich		
Groups variables	Latent/MCI	MCI/CHA	Latent/CHA	Latent/MCI	MCI/CHA	Latent/CHA	Latent/MCI	MCI/CHA	Latent/CHA
Core lexical items	ns	ns	ns	ns	*p* = 0.002	*p* < 0.001	*p* = 0.049	ns	*p* = 0.004
Proportion of core lexicon to total tokens	ns	ns	ns	ns	*p* = 0.002	*p* = 0.003	ns	ns	ns
Core lexical items per second	*p* = 0.039	ns	ns	*p* = 0.02	ns	ns	*p* = 0.02	ns	ns

#### By task

##### Cat rescue

For core lexicon total items, there were no significant differences for any group comparisons: latent aphasia and MCI groups (1.84 [CI −1.41, 5.08], *p* = 0.66), latent aphasia and CHA groups (−1.89 [−4.97, 1.18], *p* = 0.55), or MCI and CHA groups (0.06 [−2.42, 2.53], *p* > 0.99). For the proportion of core lexicon items to total tokens, there was not a significant difference for any group comparisons: latent aphasia and MCI groups (−0.01 [−0.09, 0.06], *p* > 0.99), latent aphasia and CHA groups (0.036 [−0.04, 0.11], *p* = 0.799), or MCI and CHA groups (−0.02 [−0.08, 0.03], *p* = 0.93). For core lexicon items per second, there was a significant difference between latent aphasia and MCI groups (0.24 [0.01, 0.48], *p* = 0.039), with the latent aphasia group producing fewer core lexicon items per second than the MCI group. There was not a significant difference between latent aphasia and CHA groups (−0.15 [−0.37, 0.06], *p* = 0.38) or between MCI and CHA groups (−0.09 [−0.32, 0.14], *p* = 0.94).

##### Cinderella

For core lexicon total items, there was not a significant difference between the latent aphasia and MCI groups (6.12 [−5.82, 18.06], *p* = 0.77). There was a significant difference between the latent aphasia and CHA groups (−19.18 [−28.82, −9.54], *p* < 0.001) and MCI and CHA groups (13.06 [3.26, 22.86], *p* = 0.002). The CHA group produced more core lexical items than both clinical groups (Table 5). For the proportion of core lexicon items to total tokens, there was not a significant difference between the latent aphasia and MCI groups (−0.02 [−0.097, 0.05], *p* = 0.991). There was a significant difference between the latent aphasia and CHA groups (0.101 [0.04, 0.17], *p* = 0.0003) and MCI and CHA groups (−0.08 [−0.14, −0.02], *p* = 0.002). The CHA group produced a lower proportion of core lexical items to all tokens than either clinical group. For core lexicon items per second, there was a significant difference between the latent aphasia and MCI groups (0.17 [0.02, 0.33], *p* = 0.02), but no significant differences between the latent aphasia and CHA groups (−0.01 [−0.16, 0.14], *p* > 0.99) or MCI and CHA groups (−0.16 [−0.32, 0.002], *p* = 0.055).

**Table 5:** Core lexicon items produced at least once during the Cinderella story, modeled as a percentage of the subject group (CHA, MCI, Latent) who produced them.

Core lexical item	CHA	MCI	Latent
a	100.00%	100.00%	100.00%
after	89.29%	83.33%	70.37%
all	87.50%	73.33%	66.67%
and	100.00%	100.00%	100.00%
as	78.57%	50.00%	25.93%
at	89.29%	66.67%	81.48%
away	53.57%	26.67%	22.22%
back	80.36%	60.00%	48.15%
ball	96.43%	80.00%	85.19%
be	100.00%	100.00%	100.00%
beautiful	75.00%	56.67%	29.63%
because	76.79%	56.67%	48.15%
but	91.07%	86.67%	85.19%
by	76.79%	63.33%	29.63%
Cinderella	98.21%	93.33%	92.59%
clock, o’clock	58.93%	23.33%	40.74%
come	85.71%	83.33%	70.37%
could	57.14%	43.33%	48.15%
dance	73.21%	60.00%	70.37%
daughter, stepdaughter	76.79%	53.33%	62.96%
do	92.86%	90.00%	81.48%
dress	82.14%	56.67%	66.67%
ever	89.29%	86.67%	62.96%
fairy	92.86%	73.33%	66.67%
father, dad, daddy, pa, papa	58.93%	33.33%	29.63%
find	91.07%	83.33%	55.56%
fit	89.29%	83.33%	77.78%
foot	66.07%	36.67%	29.63%
for	83.93%	73.33%	66.67%
get, got	96.43%	86.67%	92.59%
girl	62.50%	40.00%	48.15%
glass	73.21%	63.33%	33.33%
go	100.00%	100.00%	100.00%
godmother	87.50%	70.00%	74.07%
happy	89.29%	86.67%	62.96%
have	98.21%	96.67%	96.30%
he, him, his, himself	96.43%	80.00%	81.48%
home	78.57%	60.00%	37.04%
horse	60.71%	26.67%	37.04%
house	71.43%	70.00%	51.85%
I, me, my, mine, myself	75.00%	83.33%	77.78%
in	98.21%	90.00%	70.37%
into	89.29%	63.33%	44.44%
it, its, itself	94.64%	86.67%	88.89%
know	62.50%	56.67%	55.56%
leave, left	64.29%	60.00%	37.04%
like	64.29%	36.67%	51.85%
little	55.36%	40.00%	25.93%
live	94.64%	83.33%	66.67%
look	71.43%	50.00%	44.44%
lose	58.93%	50.00%	48.15%
make	82.14%	63.33%	33.33%
marry, remarry	80.36%	53.33%	62.96%
midnight	82.14%	63.33%	51.85%
mother, mom, mommy, ma, mama, stepmother	85.71%	73.33%	59.26%
mouse	67.86%	40.00%	37.04%
not	96.43%	100.00%	88.89%
of	94.64%	76.67%	88.89%
off	64.29%	36.67%	29.63%
on	89.29%	76.67%	70.37%
one	83.93%	66.67%	55.56%
out	91.07%	56.67%	51.85%
-POSS [possessive tense]	53.57%	46.67%	29.63%
prince	96.43%	96.67%	88.89%
pumpkin	78.57%	53.33%	48.15%
run	82.14%	53.33%	51.85%
say	73.21%	43.33%	55.56%
she, her, herself, hers	100.00%	100.00%	100.00%
shoe	44.64%	56.67%	51.85%
sister, stepsister	83.93%	83.33%	70.37%
slipper	92.86%	76.67%	77.78%
so	94.64%	90.00%	88.89%
strike, stroke	50.00%	30.00%	18.52%
take	62.50%	53.33%	37.04%
tell	58.93%	33.33%	33.33%
that	98.21%	96.67%	81.48%
the	100.00%	100.00%	100.00%
then	58.93%	63.33%	77.78%
there	87.50%	70.00%	59.26%
they, their, them, themselves	98.21%	96.67%	92.59%
this	76.79%	63.33%	37.04%
time	71.43%	40.00%	40.74%
to	100.00%	100.00%	100.00%
try	85.71%	63.33%	66.67%
turn	82.14%	50.00%	29.63%
two	78.57%	66.67%	85.19%
up	76.79%	73.33%	48.15%
very	71.43%	33.33%	44.44%
want	69.64%	53.33%	51.85%
well	62.50%	43.33%	55.56%
when	60.71%	33.33%	44.44%
who	76.79%	76.67%	55.56%
will	85.71%	63.33%	48.15%
with	98.21%	90.00%	88.89%

##### Sandwich

For core lexicon total items, there was not a significant difference between the MCI and CHA groups (0.95 [−1.38, 3.29], *p* = 0.92), but there was a significant difference between the latent aphasia and CHA groups (−5.26 [−9.33, −1.19], *p* = 0.004) and latent aphasia and MCI groups (4.31 [0.006, 8.61], *p* = 0.049). For the proportion of core lexicon items to total tokens, there were no significant differences identified between any groups: latent aphasia and MCI (0.03 [−0.12, 0.17], *p* = 0.999), latent aphasia and CHA (0.02 [−0.12, 0.15], *p* > 0.99) or MCI and CHA (−0.04 [−0.14, 0.05], *p* = 0.84). For core lexicon items per second, there was a significant difference between the latent aphasia and MCI groups (0.44 [0.04, 0.85], *p* = 0.02), but no significant differences between the latent aphasia and CHA groups (−0.22 [−0.50, 0.06], *p* = 0.26) or MCI and CHA groups (−0.22 [−0.62, 0.18], *p* = 0.68).

#### Core lexicon item-level group analysis

##### Cat rescue

One-way ANOVA (because data met linear assumptions) was computed to explore the impact of subject group on percentage of group members producing an item from the Cat Rescue Core Lexicon at least once (Supplementary Table 1). There was not a significant impact of group on percentage of the group that tended to produce a core lexical item (*F*[2, 99] = 1.46, *p* = 0.24, Cohen’s *f* = 0.17), suggesting that no group produced a significantly different percentage of core lexical items. This analysis was underpowered (*n* = 34 core lexicon items; achieved *β* = 0.31).

##### Cinderella

A Brown-Forsyth one-way ANOVA (because linear assumptions were not met) was computed to explore the impact of subject group on percentage of group members producing an item from the Cinderella Core Lexicon at least once. A significant main effect of group was identified (*F*[2,249.45] = 25.08, *p* < 0.0001, Cohen’s *f* = 0.42). This analysis was well powered (*n* = 94 core lexicon items; achieved *β* > 0.999). Games-Howell *post hoc* tests identified that this main effect was driven by the pairwise differences between the latent aphasia and CHA groups (0.20 [0.13, 0.27], *p* < 0.000001) and MCI and CHA groups (0.14 [0.08, 0.20], *p* < 0.000001). That is, a higher percentage of the CHA group tended to produce at least one core lexicon item at a higher percentage than the latent aphasia and MCI groups. There was not a significant difference between the latent aphasia and MCI groups (0.06 [−0.02, 0.14], *p* = 0.14).

##### Sandwich

One-way ANOVA (because data met linear assumptions) was computed to explore the impact of subject group on percentage of group members producing an item from the Sandwich procedural narrative Core Lexicon at least once (Supplementary Table 2). There was not a significant impact of group on percentage of the group that tended to produce a core lexical item (*F*[2, 72] = 0.81, *p* = 0.45, Cohen’s *f* = 0.15), suggesting that no group produced a significantly different percentage of core lexical items. This analysis was underpowered (*n* = 25 core lexicon items; *β* = 0.19).

### Aim 2: task sensitivity in identifying lexical-semantic measures differentiating the three participant groups

For only the tasks and dependent variables where there was a significant difference between two groups in one-way analyses, above, linear discriminant analysis with cross validation was used to evaluate the extent to which those metrics could classify participants into two groups (see Table 6 for prediction accuracy). The best group differentiators between the latent aphasia and MCI groups were fluency metrics, with similar prediction accuracy for core lexicon per second across all tasks. Cat Rescue (62.07%) and Cinderella (65.52%) had higher accuracy than Sandwich (55.17%). The best group differentiators between MCI and CHA groups were lexical access and diversity metrics, but only for the Cinderella task. Total core lexicon items (67.44%) and core lexicon proportion per total tokens (62.79%) had a similar accuracy for differentiating the clinical groups from the CHA group. The best group differentiators between latent aphasia and CHA groups were core lexicon items in Cinderella (78.57%) and Sandwich (80.95%), and proportion of core lexicon items in Cinderella (73.81%).

**Table 6:** Prediction accuracy percentage on unseen data (trained on 50% of data, tested on 50% unseen data).

Task	Cat rescue			Cinderella			Sandwich		
Groups variables	Latent/MCI	MCI/CHA	Latent/CHA	Latent/MCI	MCI/CHA	Latent/CHA	Latent/MCI	MCI/CHA	Latent/CHA
CoreLex					67.44%	78.57%	65.52%		80.95%
CoreLexProp					62.79%	73.81%			
CoreLexPerSec	62.07%			65.52%			55.17%		

Estimates given only for findings which were significant in one-way analyses; non-significant findings at the one-way level are blacked out.

## Discussion

### Overview of major findings

In the present study, which notably used three iterations of a single clinically accessible metric—core lexicon—showed good prediction accuracy (62–67%) in differentiating cognitively healthy adults from individuals with verified MCI using the Cinderella task (a fictional narrative). Our results also complement the recent results from Chen et al. (2024), which demonstrated the importance of evaluating the number of core lexical items produced during a familiar story narrative in differentiating between a small group of individuals with MCI from cognitively healthy adults. Prior research using ten variables extracted from discourse (a picture sequence description) has demonstrated 77% sensitivity and 80% specificity for predicting cognitive decline in individuals with cardiovascular disease (Roberts et al., 2021). Others have also found that variables derived from spoken discourse outperform data from standardized neuropsychological tests in differentiating individuals with MCI from their cognitively healthy peers (Sanborn et al., 2022). Our results also complement a review of evidence that evaluated performance of persons with MCI during picture description tasks and identified decline in semantic content (Mueller et al., 2018a). The evidence provided in our study suggests that lexical-semantic decline is indeed a noted decline in the MCI group, but that a discourse task that implicates declarative memory and produces more complex language, such as the Cinderella story, will be more sensitive than a picture description task for demonstrating subtle change in lexical-semantic decline compared to a matched cognitively healthy group. This is consistent with the amnestic presentation of the MCI cohort included in the current study.

Of particular interest was the ability of core lexicon to differentiate two groups with shared symptomology, e.g., word finding impairment and lexical-semantic degradation—those with post-stroke, latent aphasia, and those with verified MCI—which, to our knowledge, have not before been systematically differentiated. Prediction accuracy for group assignment was 65% on a procedural narrative for number of core lexicon items, and 65 and 62% on the Cinderella fictional narrative and picture description, respectively, for core lexical items per second. While the prediction accuracy in our study could be improved for differentiating these two groups, it does suggest that this is a fruitful area of future research, especially using a metric that can be ascertained in a clinical setting with relative ease.

The latent group could be differentiated from the cognitively healthy adult group with higher accuracy, for number of lexical items (78.5% on the Cinderella narrative, 81% on the procedural narrative) and proportion of core lexical items (74% on Cinderella narrative). This finding firmly refutes the clinical status quo, which is that individuals scoring above a certain criterion on established aphasia tests are not referred to or are excluded from speech and language services. Our study clearly demonstrates that, on a single metric of lexical-semantic knowledge, individuals with latent aphasia are performing worse than their cognitively healthy peers and suggests that current clinical standards reform their benchmarks in order to make services available to these individuals.

### Expanding what is known about language decline in individuals with mild cognitive impairment

Research by Toledo et al. (2018) evaluated topical propositions (e.g., main concept production) in the Cinderella narrative between cognitively healthy adults, adults with amnestic MCI, and adults with Alzheimer’s disease, but did not find a significant difference in number of propositions between the MCI and cognitively healthy control group. Similarly, a study by Drummond et al. (2015) had cognitively healthy adults, adults with amnestic MCI, and adults with Alzheimer’s disease retell a visually presented narrative, and did not find a significant difference in the number of topical propositions between the MCI and cognitively health group. Our results significantly expand upon this prior literature, suggesting that core lexicon analysis may be a more sensitive way to evaluate differences between cognitively healthy adults and adults with MCI, in both raw number of core lexicon items produced as well as the proportion of core lexicon items to total words. Interestingly, Drummond et al. (2015) also had an index – which they called the ‘discourse effectiveness index’—that reflected a similar idea to our core lexicon proportion metric. Their discourse effectiveness index was obtained by dividing the total number of words by the number of topical propositions, and this index did effectively differentiate all three of their groups (cognitively healthy, MCI, AD). This provides added support to our finding that core lexicon proportion also appeared to differentiate both the MCI and the latent aphasia group from the cognitively healthy group during a narrative task. That is, the informativeness or quality of the typical and accurate lexical-semantic information produced during a story retelling narrative has the potential to be a sensitive indicator of subtle language change in clinical groups.

When comparing the current results to prior research in MCI and AD, it is important to consider the impact of task instructions on discourse production. The majority of research in MCI and Alzheimer’s disease has used the “Cookie Theft” picture description task from the Boston Diagnostic Aphasia Examination (BDAE; Goodglass and Kaplan, 1972). Elicitation for this task instructs individuals to “tell me everything you see going on in this picture.” From a linguistic perspective, these instructions are more likely to produce discourse with limited macrostructural organization and lower coherence since there is no impetus to tell a narrative. Instead, the production is characterized by utterances with simple syntactic structures that list people, actions, and objects, with limited links between them (Wright and Capilouto, 2009). From a cognitive perspective, these instructions likely reduce cognitive effort, since development and production of a narrative requires greater contributions of attention, memory, and executive function than producing relatively independent utterances. These instructions may reduce the confound between cognitive impairments and language impairments in individuals with MCI or AD, allowing for a more precise understanding of how neurodegeneration specifically impacts language. However, these insights are limited to primarily microlinguistic features of language, given the limited macrostructural organization.

In this project, we investigated discourse produced in response to the “Cat Rescue” single picture stimulus. Elicitation for this task instructs individuals to, “Look at everything that’s happening and then tell me a story about what you see. Tell me the story with a beginning, a middle, and an end.” These instructions elicit a more complex discourse sample which includes more macrostructural organization, coherence, and cohesive ties (e.g., use of pronoun referents, temporal links, and conjunctions). As such, the individuals with MCI in this study likely experience increased cognitive demands during the picture description task than individuals in studies which use the Cookie Theft stimulus (or other stimulus with the same or similar elicitation instructions). Despite the likely increased cognitive demands of the picture description task used here, our results are consistent with other research in MCI which has found limited sensitivity of picture description tasks to differentiate cognitively healthy controls from individuals with MCI. It may be that access to the visual stimulus throughout picture description tasks provides sufficient support to overcome the mild decrements in cognition and language experienced by individuals with MCI. Indeed, the Cinderella story retell is both the longest, most complex task, and the only narrative task that does not include visual support during the retell (individuals review a wordless picture book of the Cinderella story, but it is removed prior to beginning the retell). As such, the cognitive components (in particular, declarative and working memory) are drawn upon most by the Cinderella task in comparison to the picture description task, where the visual stimulus is present throughout, and the procedural task, which involves declarative memory but which is likely scaffolded by implicit, motor memory.

### Expanding what is known about language ability in individuals with latent aphasia

The latent aphasia group was sensitively differentiated from the cognitively healthy group using core lexical item production during both Cinderella (familiar, fictional) and Sandwich (procedural) narratives, but notably, not during the Cat Rescue single picture description. In a study by Fromm et al. (2017), it was also demonstrated that the Cinderella narrative was a sensitive task for demonstrating language impairments in persons with latent aphasia. Specifically, persons with latent aphasia tended to produce fewer utterances, reduced lexical diversity, less fluent speech (words per minute), and fewer main concepts than age-similar cognitively healthy adults. Findings from the Fromm et al. (2017) study were further confirmed by DeDe and Salis (2020), who evaluated the discourse of a different group of persons with latent aphasia during the Cinderella story. They also found that the latent aphasia group differed from controls in total production (number of words) and in speech rate. Similarly, Cunningham and Haley (2020) demonstrated a significant difference in lexical diversity on the Cinderella task between a group with latent aphasia and a cognitively healthy adult group. Finally, Salis and DeDe (2022) reported that individuals with latent aphasia demonstrate longer pauses within utterances than cognitively healthy adults during the Cinderella story (although the syntactic complexity of utterances did not differentially affect pause length within utterances between the two groups), which they interpret as evidence of the mild cognitive and language impairments experienced by individuals with latent aphasia. While these studies all confirmed existing, subtle language impairments in persons with latent aphasia, none attempted to predict or classify the extent to which different discourse tasks adequately separated the latent aphasia group from the cognitively healthy group, especially using variables extracted from a single, clinically feasible measure (core lexicon). The total number of core lexical items on both Cinderella and Sandwich narratives exceeded group prediction of 78%, suggesting real potential of this measure and narrative tasks to sensitively identify subtle impairments in the latent group.

### Moving away from evaluating only single picture descriptions clinically and in research

In the current study, the Cat Rescue picture description was least successful at differentiating the three groups in one-way analyses, and subsequent supervised classification demonstrated its limited effectiveness in predicting group membership based on core lexical variables (see Tables 4, 6). Our findings add to a burgeoning literature that demonstrates the limited utility of using single picture descriptions to accurately and sensitively detect subtle language impairments, such as those demonstrated by individuals with MCI and individuals with latent aphasia, and urges researchers and clinicians to additionally collect data using tasks that draw upon macrostructural and cognitive resources, such as narratives. A recent article evaluated a large number of lexical, semantic, and syntactic variables extracted using Natural Language Processing across several monolog discourse genres in *n* = 25 cognitively healthy adults, *n* = 25 individuals with MCI, and *n* = 25 individuals with Alzheimer’s disease (Clarke et al., 2021). Findings complemented our own, in that the Cinderella narrative had the highest accuracy (0.78) and sensitivity (0.75), outperforming picture description (accuracy, 0.76; sensitivity, 0.69), procedural narrative (accuracy, 0.74; sensitivity, 0.78), conversational speech (accuracy, 0.66; sensitivity, 0.62) and novel narrative retelling (accuracy, 0.62; sensitivity, 0.53). This was likewise true when examining cognitively healthy controls versus those with MCI only, with the Cinderella narrative once again achieving the highest balanced accuracy, sensitivity, and specificity. Clarke et al. (2021) used 286 linguistic features derived via automated linguistic analyses, which is not clinically feasible, but does present evidence that using overlearned narratives, which likely draw heavily on cognitive, microlinguistic, and macrolinguistic processes, is warranted for populations with subtle language impairments.

The findings presented in this paper suggest some clinical feasibility, as well. Calculating the total core lexicon items is likely the most feasible of the three dependent variables, as the other two variables (proportion of total words and per second) involve a subsequent step, i.e., transcribing and/or recording the sample and then analyzing the sample. Since the tallying of total core lexicon items can be done “live” (Dalton et al., 2020a,b; Kim et al., 2022), and was shown to be beneficial for differentiating the latent aphasia group from the cognitively healthy adult group for both Cinderella and Sandwich, and the MCI from the cognitively healthy group for Cinderella, practitioners can uptake this practice to improve discourse assessment sensitivity when evaluating for lexical-semantic impairment.

### Future directions

A future, longitudinal, prospective design is the next step to establish the efficacy of core lexical variables in being an early identifier of cognitive decline in individuals with cerebrovascular disease and stroke. In addition, the specificity of these tasks to identify only individuals with language or cognitive changes should be determined to ensure over-referral of individuals with intact language and cognition does not occur. Finally, combination of core lexicon metrics (i.e., total, proportion and rate variables modeled together) and combination of core lexicon with other discourse-level metrics [e.g., main concept analysis (Nicholas and Brookshire, 1995)] will be imperative for identifying sensitivity (and specificity) for discourse outcomes for these populations.

### Limitations

As with many studies of individuals with aphasia and MCI, the sample included here had restricted racial and ethnic diversity. This reduces the ability to generalize these findings to diverse patients with latent aphasia or MCI. While the field of aphasiology has begun to recognize the importance of including diverse populations in research, systemic barriers continue to persist in the recruitment and retention of minoritized groups. This is particularly evident in database driven research, since the growth of a database relies upon voluntary contributions to amass a sufficiently large sample to allow for investigation.

In addition, the number of individuals with latent aphasia and MCI in the study, while generally consistent with sample sizes seen in the extant literature, may have impacted the sensitivity of the linear discriminant analysis, especially given the overlap between the three groups. Cross validation was used to ameliorate some concern. Given the promising results reported here, further investigation with larger sample sizes is warranted.

## Conclusion

In this study we demonstrated the utility of core lexicon analysis to differentiate between groups of cognitively healthy adults and adults with latent aphasia or MCI during production, with the overlearned Cinderella narrative having the most sensitivity in doing so. In sum, core lexicon analysis appears to be a sensitive, and potentially clinically feasible way to identify differences between cognitively healthy adults and individuals with mild cognitive and/or language deficits. This has the potential to increase access to rehabilitation for these individuals, thereby improving participation and quality of life.

## Data Availability

The original contributions presented in the study are included in the article/Supplementary material, further inquiries can be directed to the corresponding author.

## Footnotes

^1^ https://osf.io/je64u/
