# Supplementary material for: Access to context-specific lexical-semantic information during discourse tasks differentiates speakers with latent aphasia, mild cognitive impairment, and cognitively healthy adults
Source: Front Hum Neurosci. 2025 Jan 22;18:1500735. doi: 10.3389/fnhum.2024.1500735 (PMC11794212; doi:10.3389/fnhum.2024.1500735)
Supplement: Supplementary file 1 [file Table_1.docx]

Supplementary Material

Supplementary Table 1: Core lexicon items produced at least once during the Cat Rescue description, modelled as a percentage of the subject group (CHA, MCI, Latent) who produced them. Gray are content words (nouns; action verbs). In other words, the item “a” was produced by 82.14% of the CHA group, 92.31% of the MCI group, and 89.66% of the latent aphasia group.

| Core lexical item | CHA | MCI | Latent |
| --- | --- | --- | --- |
| a | 82.14% | 92.31% | 89.66% |
| and | 100.00% | 100.00% | 96.55% |
| bark | 60.71% | 58.97% | 75.86% |
| be | 98.21% | 100.00% | 100.00% |
| call | 71.43% | 66.67% | 48.28% |
| cat | 94.64% | 97.44% | 93.10% |
| climb | 58.93% | 64.10% | 51.72% |
| come | 91.07% | 82.05% | 65.52% |
| department | 51.79% | 56.41% | 41.38% |
| dog | 92.86% | 100.00% | 96.55% |
| down | 85.71% | 71.79% | 68.97% |
| father,dad,daddy,pa,papa | 83.93% | 89.74% | 68.97% |
| fire | 64.29% | 74.36% | 55.17% |
| fireman | 41.07% | 46.15% | 89.66% |
| get,got | 89.29% | 94.87% | 82.76% |
| girl | 83.93% | 82.05% | 48.28% |
| go | 69.64% | 66.67% | 55.17% |
| have | 71.43% | 56.41% | 75.86% |
| he,him,his,himself | 73.21% | 74.36% | 72.41% |
| in | 87.50% | 69.23% | 86.21% |
| ladder | 83.93% | 89.74% | 79.31% |
| little | 78.57% | 82.05% | 51.72% |
| not | 58.93% | 69.23% | 55.17% |
| out | 44.64% | 41.03% | 34.48% |
| she,her,herself,hers | 76.79% | 84.62% | 72.41% |
| so | 80.36% | 69.23% | 37.93% |
| stick | 48.21% | 51.28% | 100.00% |
| the | 100.00% | 100.00% | 55.17% |
| there | 48.21% | 58.97% | 55.17% |
| they,their,them,themselves | 73.21% | 71.79% | 93.10% |
| to | 100.00% | 100.00% | 96.55% |
| tree | 100.00% | 100.00% | 89.66% |
| up | 92.86% | 94.87% | 44.83% |
| with | 48.21% | 66.67% | 51.72% |

Supplementary Table 2: Core lexicon items produced at least once during the Sandwich procedural narrative, modelled as a percentage of the subject group (CHA, MCI, Latent) who produced them. Gray are content words (nouns; action verbs).

| Core lexical item | CHA | MCI | Latent |
| --- | --- | --- | --- |
| a | 80.36% | 70.00% | 65.52% |
| and | 100.00% | 100.00% | 100.00% |
| bread | 100.00% | 96.67% | 96.55% |
| butter | 100.00% | 100.00% | 100.00% |
| get,got | 75.00% | 56.67% | 65.52% |
| it,its,itself | 87.50% | 70.00% | 89.66% |
| jelly | 94.64% | 100.00% | 89.66% |
| knife | 51.79% | 53.33% | 31.03% |
| of | 94.64% | 90.00% | 86.21% |
| on | 100.00% | 96.67% | 96.55% |
| one | 69.64% | 73.33% | 72.41% |
| other | 64.29% | 73.33% | 65.52% |
| out | 53.57% | 50.00% | 34.48% |
| peanut | 100.00% | 100.00% | 100.00% |
| piece | 57.14% | 60.00% | 51.72% |
| put | 96.43% | 80.00% | 96.55% |
| slice | 67.86% | 30.00% | 37.93% |
| spread | 60.71% | 50.00% | 44.83% |
| take | 62.50% | 60.00% | 41.38% |
| the | 100.00% | 96.67% | 100.00% |
| then | 75.00% | 83.33% | 68.97% |
| to | 60.71% | 43.33% | 37.93% |
| together | 64.29% | 66.67% | 62.07% |
| two | 67.86% | 80.00% | 68.97% |
| you,your,yours,yourself | 67.86% | 76.67% | 62.07% |

Supplementary Table 3. Discourse reporting standards table. The following discourse reporting standards were developed through an expert consensus process conducted as part of a FOQUS Aphasia (www.foqusaphasia.com) initiative. These standards reflect expert opinion at the time they were developed. The authors intend for this to be a dynamic set of recommendations that will shift as the needs and practices within clinical and research communities change. For details regarding the development of these recommendations, or when using these recommendations, cite: Stark, BC & Bryant, L; Themistocleous, H; den Ouden, D-B; Roberts, A (2021). Best Practice Guidelines for Reporting Spoken Discourse in Aphasia and Neurogenic Communication Disorders. Doi: 10.1080/02687038.2022.2039372. Visit https://osf.io/y48n9/ for updates on the project.

|  | | | | |
| --- | --- | --- | --- | --- |
| Category | Item Number | Reporting Standard | Included (Mark ‘x’) | Page Number(s) |
| *Information about the discourse sample* | 1 | Define “discourse” | X | 6 |
|  | 2 | Define “utterance” (or other unit, e.g., turn unit) | n/a | n/a, not used in analyses |
|  | 3* | Number of words in sample | n/a reported on core lexicon, as main outcome | Core lexicon variables reported in Table 3 |
| *Information about how the discourse sample was collected* | 4 | Describe elicitation task | X | 16 |
|  | 5 | Exact instructions used to elicit discourse sample | X | 16 |
| *Information about the persons included in the collection of the discourse sample* | 6 | Demographic information about primary speaker [the person whose discourse is of interest] | X | Table 1 |
|  | 7 | Information about the primary speaker's neurological condition | X | Table 1; pgs 14-15 |
| *Methodology and rater agreement* | 8 | Inter-rater reliability for each analyzed variable/measure | n/a, automatic extraction | General reliability discussed on pg 16 |
|  | 9 | Reliability statistics used | n/a | n/a |
|  | 10 | Details on the number (percentage) of files used for determining reliability/agreement | n/a | n/a |
|  | 11* | Reliability (point to point agreement) for transcription (orthographic or other) | n/a | n/a |
| *Analysis* | 12 | Type of transcription (e.g., orthographic, phonetic) | X | 16 |
|  | 13 | Detailed description of any perceptual rating scale used, including providing a copy of the scale if not previously published | n/a | n/a |
|  | 14 | Details of the annotation system, formal (e.g., CHAT) or informal (created by the clinician/examiner) | X | 16-18 |
|  | 15 | Whether transcription was verbatim (e.g., including all behaviors such as fillers) or whether information was excluded in the transcription process. | X | 16 |
|  | 16 | Completeness of transcription (full, partial, transcribing errors only) | X | 16 |
|  | 17* | Details of any software used for transcribing/annotating/generating data (e.g., SALT, CLAN, ELAN) | X | 16-18 |
|  | 18* | Who/what transcribed the sample (by a human, by a machine/software, hybrid human and software) | X | 16 |
| *Information about the individual discourse variables/behaviors reported* | 19 | What is being used as primary outcome measure(s) (e.g., linguistic information, speech information, etc) | X | 16-17 |
|  | 20* | Theoretical rationale for selecting variable/behavior/outcome measure(s) | X | 10-11 |
|  | 21 | Operational definition for each variable/behavior/outcome(s) | X | 10-11 |

**Note.** Asteriks denote RECOMMENDED standards. All others are NECESSARY. Per a priori set criteria, NECESSARY reporting items are those noted as “highly” or “extremely” necessary by >70% participants in Round 3 of the expert consensus process. RECOMMENDED items are those recommendations noted as “highly” or “extremely” necessary by >65% in Round 2, but which did not reach “highly” or “extremely” necessary by >70% participants in Round 3, or those rated “highly” or “extremely” necessary by >70% in Round 1 that were not carried forward to subsequent rounds of the consensus process.
